# Function-driven single-cell genomics

**DOI:** 10.1111/1751-7915.12247

**Published:** 2015-01-28

**Authors:** Tanja Woyke, Jessica Jarett

**Affiliations:** DOE Joint Genome InstituteWalnut Creek, CA, 94598, USA

Even prior to observing the great plate anomaly, one of the most enduring questions in microbial ecology has been, ‘who is there, and what are they doing?’. While we are still far from getting a true handle on microbial diversity on this planet, the ‘who’ has become easier to address, with the rise of environmental surveys of 16S ribosomal RNA sequences in the 1990s, allowing charting of community structure in seemingly countless ecosystems. The advent of shotgun metagenomics in the early 2000s enabled the latter question to be tackled by providing access to the functional potential of uncultivated microbes (‘what *could they be* doing?’). Next-generationto be tackled to be tackled sequencing emerged shortly thereafter, and the resulting exponential increase in sequencing throughput opened the floodgates to the cultivation-independent, sequence-based exploration of the microbial world. Transitions from 16S rRNA gene diversity surveys, to metagenomics and more recently single-cell genomics (SCG), were paralleled and to a large extent fuelled by similar technological advancements in sequencing methodologies, enabling megabase-to eventually terabase- scale surveys.

Within the cultivation-independent genomics toolkit, SCG has unique advantages, as one can access the functional potential of completely unknown microorganisms without having to rely on accurate assembly and binning methods for metagenome data. One of the primary benefits of SCG is that it enables interrogation of samples at the basic unit of life, which is individual cells, allowing access to all of the nucleic acids and linkage of features like plasmids and cell-contained phages to the genome. This technology has become a standard approach, and single-cell genomes are now routinely sequenced and populate the public databases, just as metagenomes did 10 years ago. Howard Ochman's 2007 Crystal Ball prediction became reality, although isolating the single cells in the morning and obtaining their genome after lunch (‘complete, gap-free and annotated’) will still require further technology development, even 7 years later. Perhaps the continued development of single-molecule sequencing technologies such as single-molecule real-time sequencing and nanopore-based single-molecule sequencing may obviate the need to amplify a single cell's genome and eventually allow the direct sequencing of the DNA contained within an individual environmental cell, including its epigenome, thus enabling this ‘after-lunch’ single-cell genome.

While all these sequence-based methods have enabled tremendous progress in microbial ecology, they have also inadvertently given rise to an era of homology creep; one of today's key challenges is the slow pace of functional validation, which continues to lag further and further behind the rate of sequencing. Glancing into the future, it seems clear that annotating gene function and phenotype based on experimental evidence will be critical to understand this flood of environmental sequence data. Multi-omics approaches applied to communities encompassing genomics, transcriptomics, proteomics and metabolomics will greatly improve our basic understanding of microbial ecology and assist us in uncovering novel solutions for biotechnological applications. While multi-omics will be one strategy, we envision that these efforts will be heavily complemented with single-cell-based approaches. Thus, what our crystal ball reveals is the union of single-cell sequencing with an array of methodologies that enable functional or phenotypic characterization on a single-cell level prior to sequencing, and the high-throughput implementation of some of these methods.

So what exactly might such a future look like? We currently possess and are actively improving the ability to handle single-micrometre scale cells using microfluidics and microdroplets as well as the ability to generate sequencing libraries from sub-nanogram quantities of DNA; these techniques can be combined with new and evolving methods that provide functional information at single-cell resolution and are non-destructive to nucleic acids. Some methodologies for functional screening of single cells already exist, such as Fluorescent Substrate Single Amplified Genome Analysis (FS-SAGA) (Martinez-Garcia *et al*., 2012). FS-SAGA utilizes fluorescently labelled substrates of interest, such as polysaccharides, which bind to cells in a microbial population, thereby labelling the cells and enabling selective fluorescence-activated cell sorting combined with single-cell sequencing. Raman microspectroscopy can rapidly and non-destructively reveal the composition of specific molecules in a cell providing a molecular fingerprint, and can be combined with SCG, fluorescent in situ hybridization (FISH), isotope labelling and other techniques (Wagner, 2009). Activity-based protein probes can be used to label active enzymes with fluorophores or other molecules (Cravatt *et al*., 2008), allowing sorting of cells containing an enzyme of interest.

While trying to understand microbial life one cell at a time may seem rather absurd today, much like attempting to understand the universe ‘star by star,’ linking phenotypic traits and sequence information on the single-cell level, in conjunction with multi-omics approaches and across different spatial scales, will certainly bring us one step closer to understanding microbial communities and complex microbial systems. Importantly, it will allow us to zoom in on a biological or biogeochemical process of interest and capture the cells that are involved in this process, separating them from their often complex community. The most exciting potential that function-driven SCG has to offer relates to uncovering the ‘unknown unknowns’. An example of such ‘unknown unknowns’ is the recent discovery that the candidate phylum ‘Tectomicrobia’ is involved in the synthesis of bioactive metabolites previously thought to be rather limited in taxonomic range (Wilson *et al*., 2014).

In our view, now and in the next several years to come, function-driven SCG will be another powerful genomics tool along with next-generation sequencing technologies, providing a glimpse into more than ‘just’ the coding potential of the microbial world. Eventually, this synthesis of sequence and functional information will move us from mere description to unifying theories and predictions in microbial ecology, called for by Tom Curtis in his 2007 Crystal Ball.

## Conflict of interest

None declared.
